# Molecular Cloning and Characterization of cDNA Encoding a Putative Stress-Induced Heat-Shock Protein from *Camelus dromedarius*

**DOI:** 10.3390/ijms12074214

**Published:** 2011-06-27

**Authors:** Mohamed S. Elrobh, Mohammad S. Alanazi, Wajahatullah Khan, Zainularifeen Abduljaleel, Abdullah Al-Amri, Mohammad D. Bazzi

**Affiliations:** Genomic Research Chair Unit, Department of Biochemistry, College of Science, King Saud University, PO Box 2455, Riyadh 11451, Saudi Arabia; E-Mails: msanazi@ksu.edu.sa (M.S.A.); wkhan@ksu.edu.sa (W.K.); zarifeen@ksu.edu.sa (Z.A.); abdullah@KSU.EDU.SA (A.A.-A.) mbazzi@ksu.edu.sa (M.D.B.)

**Keywords:** Arabian camel, molecular cloning, HSPA6, sequence characterization, cDNA cloning, 3D structure, alignment, RACE, real-time PCR

## Abstract

Heat shock proteins are ubiquitous, induced under a number of environmental and metabolic stresses, with highly conserved DNA sequences among mammalian species. *Camelus dromedaries* (the Arabian camel) domesticated under semi-desert environments, is well adapted to tolerate and survive against severe drought and high temperatures for extended periods. This is the first report of molecular cloning and characterization of full length cDNA of encoding a putative stress-induced heat shock HSPA6 protein (also called HSP70B′) from Arabian camel. A full-length cDNA (2417 bp) was obtained by rapid amplification of cDNA ends (RACE) and cloned in pET-b expression vector. The sequence analysis of *HSPA6* gene showed 1932 bp-long open reading frame encoding 643 amino acids. The complete cDNA sequence of the Arabian camel *HSPA6* gene was submitted to NCBI GeneBank (accession number HQ214118.1). The BLAST analysis indicated that *C. dromedaries HSPA6* gene nucleotides shared high similarity (77–91%) with heat shock gene nucleotide of other mammals. The deduced 643 amino acid sequences (accession number ADO12067.1) showed that the predicted protein has an estimated molecular weight of 70.5 kDa with a predicted isoelectric point (pI) of 6.0. The comparative analyses of camel HSPA6 protein sequences with other mammalian heat shock proteins (HSPs) showed high identity (80–94%). Predicted camel HSPA6 protein structure using Protein 3D structural analysis high similarities with human and mouse HSPs. Taken together, this study indicates that the cDNA sequences of HSPA6 gene and its amino acid and protein structure from the Arabian camel are highly conserved and have similarities with other mammalian species.

## 1. Introduction

All organisms react to extreme environmental factors through *de novo* as well as by way of considerable elevation in several gene expressions associated with cell protection from the adverse effects of intracellular protein denaturation. These genes encode for a family of heat shock proteins (HSPs) including other molecular chaperones and cytoprotective proteins. Severe physical stimuli and chemical contaminants result in elevated expression of HSPs which play a crucial role under variable stress conditions [1–3]. As molecular chaperones, HSPs are in charge of maintaining the correct folding and shielding various proteins from a number of factors by restoring their native structures [4]. The inducible forms of the HSPs due to a variety of stresses result in elevated level of transcription and translation compared to the normal conditions to guard against stress-inducing agents [5]. Surrounding temperatures have enormous effect on heterothermic organisms including their biological and physiological functions at a molecular level [6]. Interestingly, because the mammalian heat shock response is a very much conserved process, it plays a key role in heat-induced cell killing protection. Extreme temperature has great influence on molecular and biochemical processes; hence HSPs have largely been utilized as biomarkers for biotic and abiotic stresses [7,8]. The most studied isoform among HSPs is the HSP70 in relation to chemical and environmental stresses [5]. *HSPA6* (also called *HSP70B*′), a co-chaperone gene encoding heat shock protein 6 (70 kDa), has been implicated in chaperoning the network which support cellular proteostasis [9]. HSPA6 induction has been exploited as a reasonable biomarker of cellular stresses [10–12] and its mRNA expression levels have been shown to be significantly induced against a variety of cellular stresses [13–15]. HSP70B′ is evolutionarily closely related to human HSP72 and is suggested to play cooperative roles in cell survival of proteotoxic stress. Unlike HSP72, HSP70B′ (HSPA6) is strictly inducible, having no noticeable basal level of expression in most cells [10,16], however interestingly no homologs of rodent HSP70B′ gene have been found [16].

The Arabian or one-humped camel (*Camelus dromedarius*), belongs to the family Camelidae that has six camel-like animals (one hump and two hump camels, llama, alpaca, guanaco, and vicuña), and is found in the Arabian deserts and arid and semi-arid areas of the Middle-East [17]. The Arabian camel, domesticated under semi-desert environments developed unique physiological modifications to acclimatize with stressful desert conditions such as high temperature and drought. Compared to small and large true ruminants; the mortality rate in adult camels is very low in the event of drought conditions [18]. Like other land animals, the Arabian camel resorts to selective cooling of heat stress conditions, hence protecting the heat-sensitive brain tissue from stress and shows enhanced tolerance against high temperatures [19]. Such responses are essential for normal physiological functions [20] and one of the first physiological roles linked with stress-induced increase of the inducible HSP70 was acquired thermotolerance [21].

Keeping in mind the habitat and physiological conditions in which the Arabian camel survives, the objective of the present study was to clone and characterize a full length cDNA encoding a putative stress-induced heat shock protein from the Arabian camel. In addition, the obtained cDNA sequences were compared with those of other mammalian species. The amino acid sequences of HSPA6 were also compared with other mammalian species. This is the first report that deals with the cloning and characterization of cDNA encoding a putative stress-induced heat shock protein from the Arabian camel.

## 2. Results

### 2.1. Characterization of HSPA6 Gene Full-Length cDNA

**Figure 1:**
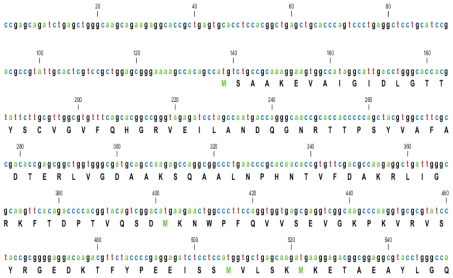

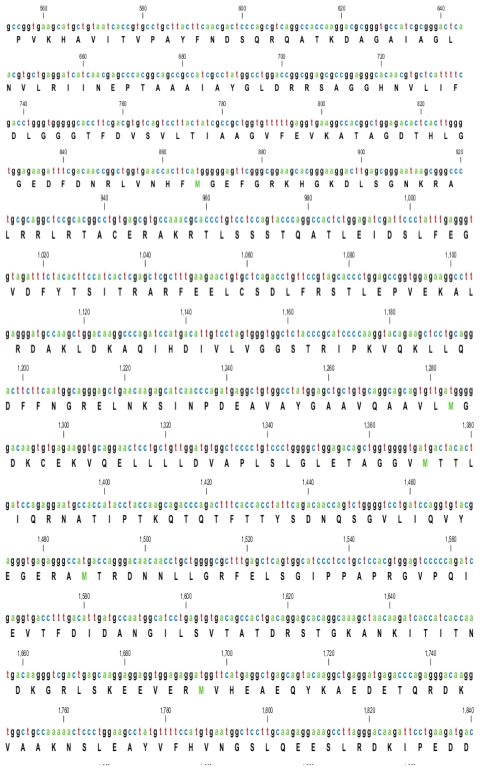

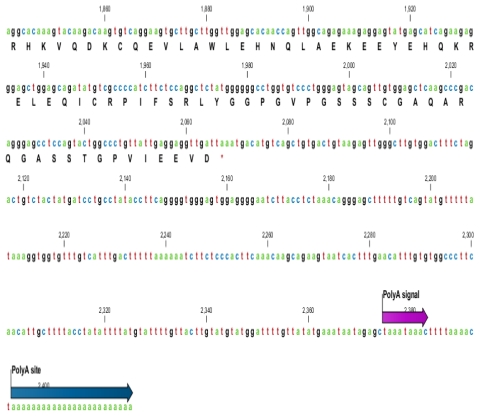
Complete nucleotide sequence submitted to NCBI GeneBank (accession number HQ214118.1) encoding a camel putative *HSPA6* gene and its corresponding 643 amino acid sequences (Database accession number ADO12067.1). The initiation codon (atg) and end codon (taa) are shown at position 137 bp and 2067 bp, respectively. The poly (A) tail signal peptide and poly (A) tail is shown at positions 2375 and 2394 bp respectively.

Based on sequence homology, the designed primers amplified a single 0.5 kb fragment which, when sequenced, showed homology to other mammalian *HSPA6s*. Another set of primers were then used to amplify the whole *HSPA6* fragment which amplified a 1987 bp fragment corresponding to the 5′ end of the gene but was still missing the 3′ end. In order to get the 3′ end, we used the RACE technology from MCLAB, USA and successfully obtained a full-length cDNA fragment of 2417 bp by rapid amplification of cDNA ends (RACE). The sequence analysis showed 1932 bp long open reading frame of *HSPA6* gene encoding 643 amino acids and consisted of 136 bp and 349 bp corresponding to the 5′- and 3′-terminal UTR (untranslated regions) respectively (Figure 1 and Table 1).

The BLAST analysis showed that *C. dromedaries HSPA6* nucleotides shared high similarity (77–91%) with heat shock gene nucleotide from other mammals (human, orangutan, horse, cow, pig, mouse, dog, and panda) (Table 1). The entire nucleotide sequence of camel *HSPA6* gene shows 91% homology with cow and pig; 89% with human, orangutan and horse; and 81%, 80% and 77% homology with panda, dog, and mouse respectively; indicating a close evolutionary relationship.

### 2.2. Protein and RNA Secondary Structure Prediction

**Figure 2:**
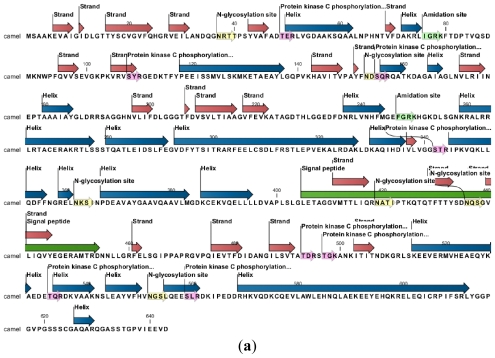

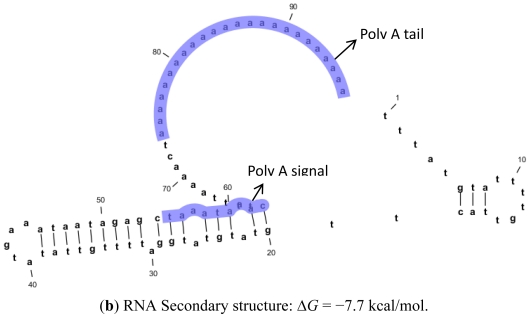
(**a**) The camel heat shock protein’s motifs secondary structure annotation site prediction show its *N*-glycosylation, protein kinase *C*-phosphorylation sites along with secondary structure prediction, amino acid distribution and prediction of hydrophobicity; (**b**) Predicted RNA secondary structure of camel HSP gene, arrows showing structural motifs of poly (A) tail and signals.

**Figure 3:**
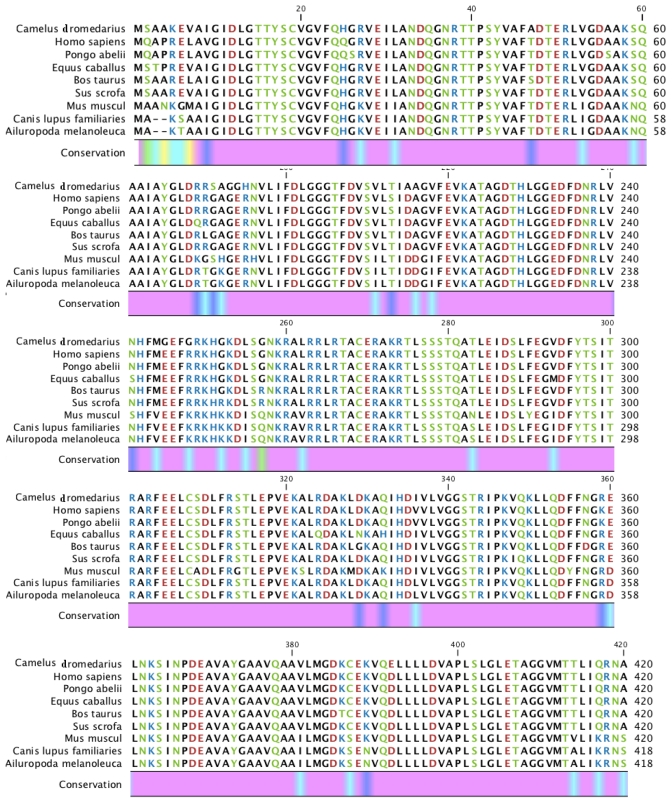

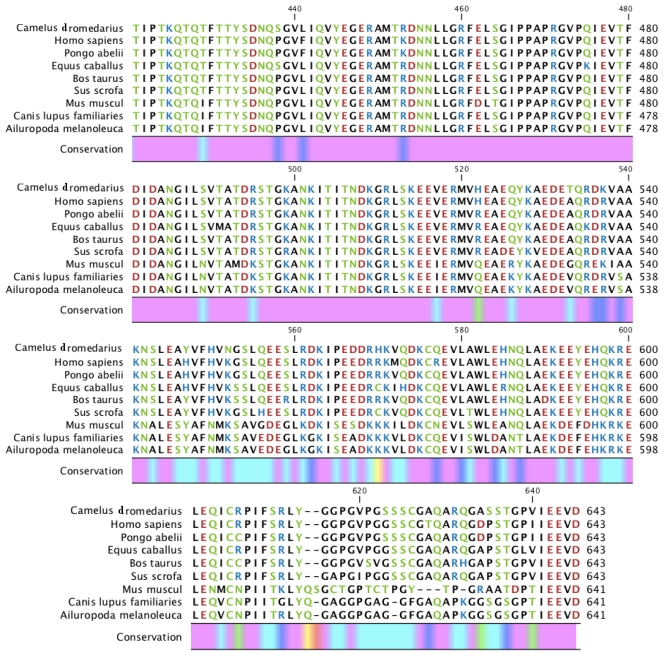
Multiple sequence alignment of amino acid sequences of the Arabian camel HSPA6 (GenBank accession number ADO12067) with eight mammalian species human (NP_002146), orangutan (XP_002809930), horse (XP_001488189), cow (XP_589747), pig (NP_001116599.1), mouse (AAA74906), dog (BAC79353), giant panda (XP_002931115). Identical residues are shaded in pink color.

The RNA secondary structure using CLCbio free Genome workbench (CLC Genome workbench v6.0.1) with the free energy minimization algorithm showed a 24 bp-long poly (A) tail and 9 bp long signal from Camel *HSPA6* mRNA sequence (Figure 2). The complete cDNA sequence of the Arabian camel *HSPA6* was submitted to NCBI GeneBank (accession number HQ214118.1). The deduced 643 amino acid sequences (Database accession number ADO12067.1) showed that the predicted protein has an estimated 70.5 kDa molecular weight (Table 2). The comparative analyses of camel HSPA6 protein sequences with other mammalian (human, orangutan, horse, cow, pig, mouse, dog, and panda) HSPs available from GenBank showed high identity (80–95%), with an isoelectric point (pI) of 6.0. (Table 2 and Figure 3). The predicted amino acid sequence of 643 residues of the Arabian camel *HSPA6* gene was 94% similar to human, orangutan, cow and pig; 93% to horse; whereas 80% and 83% to mouse and dog respectively (Table 2). However, the predicted molecular weight and isoelectric point did not differ much from other mammalian HSPs (Table 2).

### 2.3. Phylogenetic Analysis

**Figure 4:**
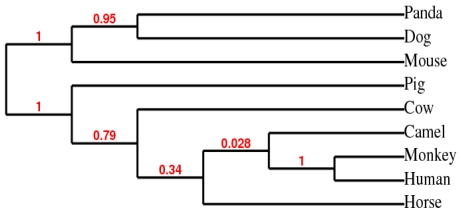
Phylogenetic relationship of the Arabian camel HSPA6 amino acid sequences (GenBank accession number ADO12067) with amino acid sequences from eight mammalian species using PhyML 3.0 aLRT Phylogeny based on maximum likelihood algorithm.

The phylogenetic tree inferred from amino acid sequences of HSPs from all nine mammalian species was based on maximum likelihood algorithm using CLCbio Genome workbench (Figure 4). The panda (XP_002931115), dog (BAC79353) and mouse (AAA74906) HSPs appeared to be distinct from the camel HSPA6 (ADO12067) which grouped closely with human (NP_002146), orangutan (XP_002809930), and horse (XP_001488189) and then with cow (XP-589747) and pig (NP_001116599.1).

### 2.4. Camel HSPA6 Protein 3D Structure Prediction

**Figure 5:**
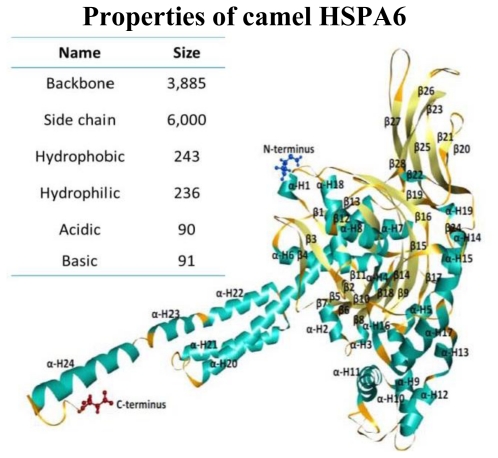
The Arabian camel HSPA6 predicted 3D structure based on multiple threading alignments by LOMETS and interactive TASSER assembly simulations showing C- and N-terminal region along with α and β residues showing a total of 29β sheet and 24 α-helix in the predicted structure.

**Figure 6:**
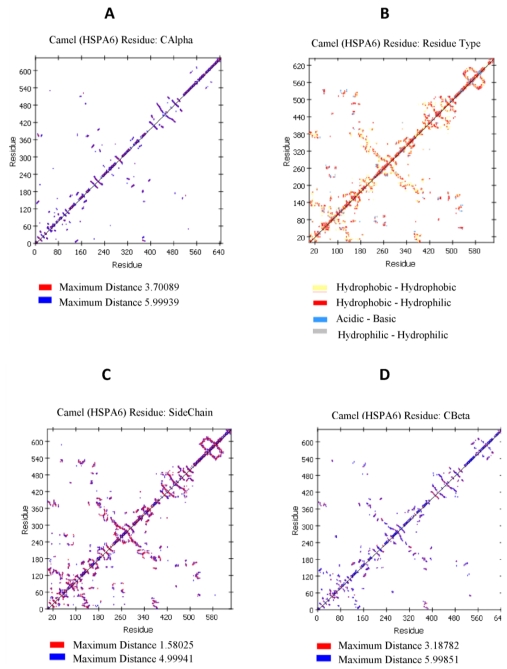
Protein molecular modeling and data analysis for camel heat shock protein (HSPA6) were performed using Accelrys Discovery Studio Visualizer for (**a**) C-α residue to residue distance; (**b**) residue type interactions such as Hydrophobic-Hydrophobic, Hydrophilic-Hydrophilic, Acidic-Basic; (**c**) residue side chain and (**d**) residue for C-β.

**Figure 7:**
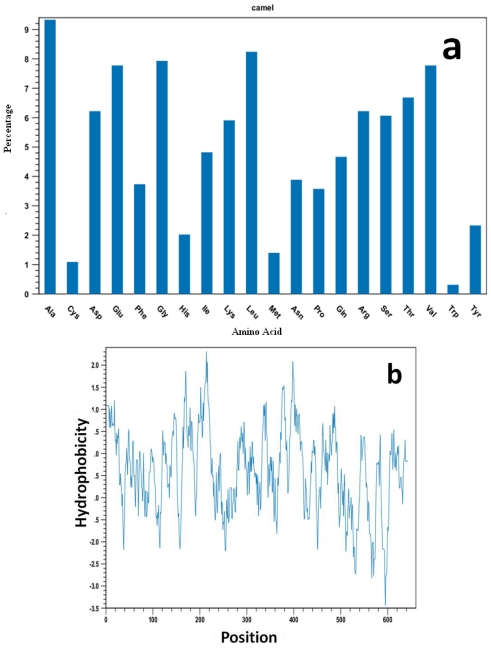
(**a**) Amino acid distribution percentage and (**b**) hydrophobicity plot for the Arabian camel HSPA6. The positions of amino acid residues are numbered beginning with the first methionine.

Camel HSPA6 3D protein crystal structural model was predicted from amino acid sequences using predict ITASSER sever based on multiple-threading alignments by LOMETS and iterative TASSER assembly simulations to get the most precise 3D protein structural prediction (Figure 5). The 3D crystal structural model consisted of 24 α-helices and 29 β-sheets in the predicted structure while the backbone consisted of 3885 where the side chain was 6000. The sizes of the hydrophobic and hydrophilic residues were 243 and 236 amino acids, respectively (Figure 6). The acidic and basic residues were 90 and 91 amino acids, respectively. The Arabian HSPA6 protein’s amino acid distribution and prediction of hydrophobicity analysis are shown in Figure 7a,b, respectively. The distribution of amino acids from the camel *HSPA6* cDNA sequences showed a high percentage (>9%) of alanine (Ala) and lowest (<1%) percentage of tryptophan (Trp). The hydrophilicity plot is a type of quantitative analysis to assess the degree of hydrophobicity or hydrophilicity of amino acids of a protein and can be utilized to distinguish possible structure or domains of a protein.

### 2.5. Camel HSPA6 3D Crystal Structures Alignment with Other Mammalian Species

**Figure 8:**
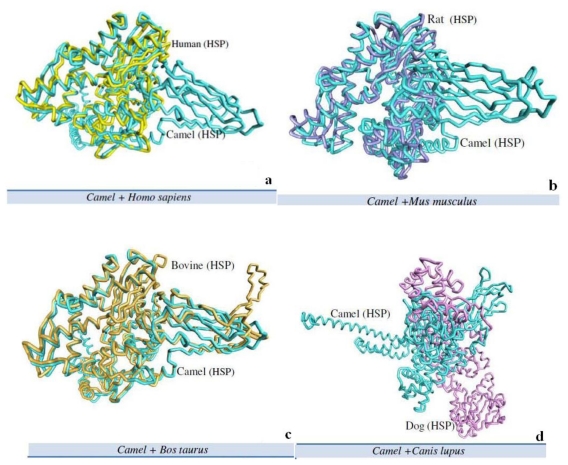
The structure of camel HSPA6 3D aligned with four mammalian species’ HSPs. The Arabian camel HSPA6 3D protein structure was aligned with crystal structure of other mammalian HSPs by using predict DaliLite server for Pairwise comparison between the two proteins structure; (**a**) camel with human; (**b**) camel with dog; (**c**) camel with cow and camel with mouse. The heuristic algorithm was used to illustrate and depict random structural alignments of proteins with various folds with good accuracy.

The Arabian camel HSPA6 3D protein structure was aligned with crystal structure of other mammalian HSPs by using predict DaliLite server by Pairwise comparison of two proteins structures at a time (Figure 8, Table 3 and Table 4). We have compared the 3D protein crystal structure with four other mammalian protein structures *i.e.*, human (Figure 8a), mouse (Figure 8b), cow (Figure 8b) and dog (Figure 8b). The heuristic algorithm was used to show the random structural alignments of proteins with different folds with reasonable accuracy by an extreme value distribution (Table 3). The camel HSPA6 protein structure via protein 3D structural analysis illustrated 87% and 82% resemblance with human and mouse HSPs, respectively.

## 3. Discussion

In this study, we provide the first report on the full-length cDNA and deduced protein sequence of a putative stress-induced HSPA6 from the one-humped Arabian camel that survives in desert conditions *i.e.*, extreme drought and high temperature. Heat shock proteins are associated with cytoprtoection against several stresses in mammalian species. The HSPA6 protein is strictly inducible with no detectable basal expression [22]. The HSPA6 protein is nuclear and cytosolic, its activation is considered as a suitable biomarker indicative of cell stress; and can be synthesized momentarily in response to heat stresses [15]. Because HSPA6 is evolutionarily highly related to human HSP72, they are believed to function in a similar manner; however direct role of its function in stressed cells is not known. Camel is subjected to harsh environmental conditions like high temperature and dehydration. Hence, the cytoprotection (thermotolerance) is associated with increased accumulation of HSPs, predominantly the HSP70 family. Their main role is to protect proteins from irreversible damage. Both constitutive and inducible isoforms of Hsp70 family are present in human. The two major inducible isoforms are HSP70/HSP72 (coded by *HSPA1A* gene), and HSP70B′ (coded by *HSPA6* gene). Both isoforms differ in terms of their regulation and expression. The HSP72 has a substantial basal level in many cells, whereas HSP70B′ (HSPA6) is inducible and builds up rapidly in response to stress conditions; nevertheless the HSP72 levels remain noticeable for several days [15].

A full-length cDNA of *HSPA6* was obtained through 3′ and 5′ RACE PCR and were assembled into a 2417 bp contig, cloned in pET15b expression vector. The gene putatively encodes a 1932 bp long open reading frame encoding 643amino acids and comprised of 136 bp and 349 bp 5′- and 3′-terminal untranslated regions respectively, along with a 24 bp long poly (A). The camel heat shock protein’s motifs secondary structure annotation showed N-glycosylation, protein kinase C-phosphorylation sites along with secondary structure prediction, amino acid distribution and regions of hydrophobicity. The sequence was submitted to NCBI GeneBank (accession number HQ214118.1). The BLAST indicates that the Arabian camel *HSPA6* gene nucleotides is highly conversed and shared a high similarity (up to 91%) with heat shock protein nucleotide of other mammals (human, orangutan, horse, cow, pig, mouse, dog, and panda). The highest nucleotide sequence identities of camel *HSPA6* gene were observed with cow and pig (91%); as well as with human, orangutan and horse (89%) indicating a close evolutionary relationship. High similarities with other HSP proteins have recently been studied with HSP70 gene from goat (*Capra hircus*) [23], where they observed maximum identity with cow HSP70 (97.8%). *HSPA6*, the inducible form among *HSP70* genes, is rather conserved in the mammalian lineage, with homologs reported in cotton top-tamarin *(Saguinus Oedipus*), pig (*Sus scrofa*), cow (*Bos Taurus*), and human (*Homo sapiens*) [15].

The RNA secondary structure camel *HSPA6* showed a 24 bp long poly (A) tail and 9 bp long signal based on the mRNA sequence. The 643 amino acid sequences (Database accession number ADO12067.1) suggest that the predicted protein has a molecular weight of 70.5kDa and isoelectric point (pI) of 6.0. When compared with other mammalian HSPs available from GenBank the camel HSPA6 amino acid sequences demonstrated a high identity (up to 94%). Among the eight mammalian HSP used to compare with the Arabian camel HSPA6 amino acid sequences maximum identity was shown by human, orangutan, cow and pig (94%) along with horse (93%). Gade *et al.* [23] have reported that the goat HSP70 amino acid sequences showed up to 99% similarities with cow and almost 98% with human, pig and horse.

Our results show that the Arabian camel’s HSPA6 nucleotide and amino acid sequences are highly conserved and show high similarities with other mammalian species. The HSPs are known to be highly conserved for their coding as well as regulatory sequences [24] and including those that are stress-induced HSPs [25]. The heat shock protein of dog has shown similarities with cow, human and mouse HSPs ranging from 90 to 95% [26]. Even HSPs proteins from non-mammalian species are reported to be highly conserved with mammalian species for example. The HSP gene from chicken showed 80% identity with the human cDNA sequence as well as with the amino acid sequence [27].

The phylogenetic tree based on the amino acid sequences of HSPs from all nine mammalian species using maximum likelihood algorithm showed that the camel HSPA6 (ADO12067) has a close similarities with human (NP_002146), orangutan (XP_002809930), and cow (XP-589747) but showed dissimilarities with pig (NP_001116599.1), mouse (AAA74906), dog (BAC79353) and panda XP_002931115. The protein sequences of HSP70 family members are conserved throughout evolution and show over 75% of sequence homology [28]. Regardless of such reasonably high evolutionary conservation, many HSP70 isoforms are species or cell/tissue specific [25,29–34]. The species-specific differences are most likely due to variations in thermal tolerance [35] and among species, Hsp70 isoforms may vary with regard to thermotolerance [36].

Heat shock proteins play important roles as molecular chaperones and their sequences play important roles during protein folding and transport. The predicted 3D protein crystal structure of camel HSPA6 amino acid sequences based on multiple-threading alignments by LOMETS and iterative TASSER assembly simulations showed 24 α-helices and 29 β-sheets with comparable number of acidic and basic residues where as the backbone consisted of 3,885 and the side chain was 6000. The hydrophobic were 243 and hydrophilic 236 residues long. The acidic and basic residues respectively have 90 and 91 amino acids. The HSPA6 protein’s amino acid distribution and prediction of hydrophobicity showed presence of a high percentage (>9%) of alanine (Ala) and lowest (<1%) percentage of tryptophan (Trp). Such hydrophilicity plots are quantitative measures to examine the degree of hydrophobicity or hydrophilicity of amino acids in a protein and can be used to differentiate segments or domains of a protein.

The protein 3D structural analysis of camel HSPA6 exhibited 87% and 82% similarity with human and mouse HSPs 3D structures, respectively. The HSPs are very closely related evolutionarily and share high protein sequence homologies [37]. It has been reported that some HSPs share up to 100% sequence homology in the peptide binding domains, whereas HSPA6 (Hsp70B′) homologs have been reported in other mammalian species including humans [38]. Furthermore, under heat stress conditions, HSP70B′ (HSPA6) may interact with many other proteins, chaperones, and co-chaperone proteins involved in protein folding, stabilization, and shuttling functions in the cell. HSPs can be activated by stress responses such as sub-lethal heat stress, radiation, heavy metals, ischemia, nitric oxide radicals, certain chemotherapeutics, and other stimuli that are capable of triggering heat shock transcription factors [15,38].

The HSPA6 in the Arabian camel perhaps play comparable roles similar to the other reported HSPA6 proteins to cope with the stressful conditions. In addition, such proteins are believed to show pleiotropic effects, where they performed multifactorial tasks to maintain the homeostatic regulation of the animal. With regards to the Arabian camel’s surroundings, the heat shock proteins from camel may be exploited as a model system to address unknown questions about the ecological, evolutionary and functional roles of heat shock proteins together with the regulation of their expression. Overall, this research showed that the cDNA sequences of *HSPA6* gene, its amino acids and protein structure from the Arabian camel are well conserved and have similarities with other mammalian species. Although this paper provides detailed information about HSPA6 sequences and its structure; however, more detailed studies are required to explore the role of camel HSPA6 in other gene regulation, function, response to environmental change, and their action at the molecular level.

## 4. Experimental Section

### 4.1. Sample Collection

The camel liver tissues from freshly sacrificed young adult male were collected (approximately 15–35 min after scarification) from a local modern slaughterhouse in Riyadh, officially supervised by trained veterinarians. The tissues after collection were cut with sterile scalpel in ~1 cm^3^ cubes and immersed in RNA Later Tissue Protect Tubes (Qiagen, cat no 76154) and were then kept at −80 °C until further use.

### 4.2. RNA Isolation and cDNA Synthesis

Total RNAs were extracted from camel liver tissues by transferring the collected tissue samples to another clean sterile tube containing 600 μL of RLT buffer/30 mg of liver tissue (RNeasy Mini Kit; Qiagen cat no 74104). The liver tissue samples were then homogenized using Dispomix ^®^Technology, gentleMACS ^™^ Dissociator (MACS Miltenyi Biotec). Following the RNeasy Mini Kit protocol, the RNA yield (1 μg/μL) was determined by NanoDrop 8000 (Thermoscientific™). Then, 5 μL of isolated RNA was used for reverse transcription using ImProm-II ^™^ Reverse Transcription System (Promega, cat no TM236) and Olig (dT)_15_ primers protocol. The 20 μL reaction mixture contained 5 μg of total RNA, 0.5 μg of oligo dT primer (16–18 mer), 40 U of Ribonulease inhibitor, 1000 μM of dNTP mix, 10 mM of DTT, and 5 U of MuMLV reverse transcriptase in 5× reverse transcriptase buffer. The reaction mixture was gently mixed, incubated at 37 °C for 1 hour; afterwards the reaction was stopped by heating the mixture at 70 °C for 10minutes and chilled on ice.

### 4.3. Synthesis and Confirmation of Partial cDNA of HSPA6 Gene

The *HSPA6* primers were designed based on conserved sequence homology from five mammalian species *i.e*., human, bovine, dog, monkey and pig available from National Center for Biotechnology Information (NCBI) for initial screening. The two sets of primers used for initial screening and obtaining partial *HSPA6* cDNA of camel were (1) Forward 5′ACCAGGTGGCCATGAACCCCCAGAACACCG3′ and Reverse 5′GGTTAATGCTCTTGTTGAGCTCCC 3′ and (2) Forward 5′ TCCCGCAACTGGATAAAAAG 3′ Reverse 5′ ATCGACCTCCTCGATGACAG 3′. The 50 μL PCR reaction contained 2 μL of template cDNA with 5 μL (10 μM) of each primers set, 25 μL FideliTaq ^™^ PCR Master Mix (2×) (USB, cat no 71182) and 13 μL of ultrapure Millipore water in a PCR reaction (GeneAmp ^®^ PCR System9700 and Veriti ™ Thermal Cycler, Applied Biosystems). The PCR amplification conditions were as follows; initial denaturation at 95 °C for 5 min followed by 30 cycles at 94 °C for 1min, 50 °C for 1 min and 72 °C for 2 min. Final extension step was carried out at 72 °C for 5 min. The products of PCR reaction was electrophoresed on 0.7% agarose (GE Healthcare, cat no 17-0554-03) gel using 1 kb DNA Ladder (Promega, cat No G5711). The 1kb fragment from PCR reaction was eluted and purified using QIAquick Gel Extraction kit (Qiagen, cat No 28706). Purified fragment was quantified and validated on agarose electrophoresis before sequencing. Sequencing of PCR fragments was done by chain termination sequencing using DYEnamic ET terminator kit (GE Healthcare, cat no US81090). The sequencing reactions were purified using DyeEx 2.0 Spin Kit (Qiagen, cat no 63206) and were then sequenced on MegaBace1000 Sequencing machine (GE Healthcare MegaBace1000). The samples were also sent to Functional Genomics and Proteomics Unit at the University of Birmingham, UK for further sequence confirmation.

### 4.4. Rapid Amplification of cDNA Ends (RACE), Cloning and Sequencing

*R*apid *a*mplification of *c*DNA *e*nds (RACE)-PCR was used to identify and to isolate the 5′-end and 3′-end of *HSPA6* using the MCLAB service, California, USA, according to the method of Chenchik *et al.* [39]. Camel total RNA was annealed with 5′-end and 3′end adaptor primers, and reverse transcribed respectively to tailing to respective 5′ and 3′-cDNA. The resulting single-stranded 5′ and 3′-cDNA were then used as templates in PCR. For 5′RACE, the 5′-end adaptor primer (forward primer) and *HSPA6* 5R (reverse primer 5′CCAATCAGCCTCTTGGCGTCGAACAC3′) was used. For 3′RACE, *HSPA6* 3F primer (forward primer 5′TTGAGCTCAGTGGCATCCCTCCTGCT3′) and the 3′-end adaptor primer (reverse primer) were used. The cycling program was set for five cycles of 94 °C, 5 min; 5 cycles of 94 °C 15 s, 70 °C 15 s, 72 °C 3 min; 5 cycles of 94 °C 15 s, 68 °C 15 s, 72 °C 3 min; 5 cycles of 94 °C 15 s, 65 °C 15 s, 72 °C 3 min; 25 cycles of 94 °C 15 s, 60 °C 15 s, 72 °C 3 min; 1 cycle of 72 °C, 5 min. The RACE-PCR products were purified and cloned into pCR Blunt II vector (Invitrogen) to confirm the sequences of the 5′-end and 3′-end, respectively. The full-length cDNA PCR product was cut with the restriction enzymes (*Bam*HI and *Nde*I), purified, and ligated with T4 DNA ligase into a *Bam*HI/*Nde*I-cut pET15b vector. Competent *E. coli* DH5α cells were transformed with the plasmids and selected by means of antibiotic resistance. The sequence of the inserted full-length cDNA was confirmed with DNA sequencing.

### 4.5. Analysis and Alignment of cDNA Sequence

The sequence of camel *HSPA6* gene was analyzed for protein translation, sequence alignment and comparisons with other mammalian species. The predicted protein was done by expasy translate tool [40] and Bioedit [41]. DNA and protein homologies were done using NCBI BLAST program [42] via NCBI web-server.

### 4.6. Protein and mRNA Secondary Structure Prediction

Camel *HSPA6* mRNA sequence was used to construct the secondary structure using CLCbio free Genome workbench (CLC Genome workbench, version 6.0.1, [43]) with the free energy minimization algorithm. RNA tertiary structures was characterized by secondary structural elements based on hydrogen bonds within the molecule that form several recognizable “domains” of secondary structure like stems, hairpin loops, bulges and internal loops. A poly (A) tail and poly (A) signal for the camel *HSPA6* mRNA was predicted based on free minimization algorithm of CLCbio. The predicted molecular weight, pI and charges of the protein HSPA6 were estimated [44].

### 4.7. Phylogenetic Analysis

A phylogenetic tree was constructed using amino acid sequences of HSPs from all nine mammalian species by PhyML 3.0 aLRT phylogeny based on maximum likelihood algorithm [45]. This is a simple, fast and accurate algorithm to estimate large phylogenies by maximum likelihood and quite powerful, and robust to certain violations of the model assumptions. The approximate likelihood-ratio test (aLRT) was utilized within the algorithm used by the recent fast maximum likelihood tree estimation program PHYML [45–48]. The CLCbio Genome workbench was used for multiple sequence alignment purpose for phylogenetic tree prediction based on maximum likelihood algorithm.

### 4.8. Protein Structure and Sequence Analysis

The Arabian camel heat shock protein’s motifs secondary structure annotation site prediction with its *N*-glycosylation, protein kinase *C*-phosphorylation sites along with secondary structure prediction, amino acid distribution and prediction of hydrophobicity were performed according to Eisenberg *et al.* [49] using Accelrys Discovery Studio Visualizer v3.0 for protein molecular modeling and data analysis [50,51]. We have also determined the C-α residue to residue distance; residue type interactions such as Hydrophobic-Hydrophobic, Hydrophilic-Hydrophilic, Acidic-Basic; residue side chain and residue for C-β for camel heat shock protein (HSPA6).

Multiple sequence alignment of amino acid sequence of camel heat shock protein HSPA6 with eight other mammalian species HSPs sequences were performed by using CLC Genome workbench version 6.0.1 [43]. Amino acid sequences were based on progressive alignment algorithm [52] in order to create multiple alignments.

### 4.9. Camel HSPA6 Protein 3D Structure Prediction

Camel HSPA6 structure was predicted from the amino acid sequences using predict ITASSER server based on multiple-threading alignments by LOMETS and iterative TASSER assembly simulations; the server aimed to provide the most accurate structural predictions using state-of-the-art algorithms [53,54]. We performed the model quality assessment component of protein structure prediction using the QMEAN server which provided two scoring functions based on analyzing eight round of the community-wide blind test assessed in the CASP experiment (Critical Assessment of Techniques for Protein Structure Prediction) [55,56]. The accuracy of this model is quite good for specific applications such as in the course of protein structure prediction where a vast number of models are generated and needed to choose the best candidates based on an energy function.

### 4.10. Camel HSPA6 3D Crystal Structures Alignment with Other Mammalian Species

The Arabian camel HSPA6 protein 3D structure was aligned with the crystal structure of other mammalian HSPs using predict DaliLite server with a pairwise comparison between two proteins structures at a time. The heuristic algorithm was used to describe random structural alignments of proteins with different folds and reasonable accuracy [57].

## 5. Conclusions

We believe that the HSPA6 in the Arabian camel probably play similar roles to the other reported HSPA6 proteins and assist the animal in tolerating and alleviating the stressful conditions that they live in. Furthermore, these proteins have also been suggested to have pleiotropic effects and work in coordination with multiple systems in various ways to manage the homeostatic regulation of the animal. Considering the Arabian camel’s habitation, the heat shock proteins from camel may be utilized as a model system to address unknown questions regarding the ecological, evolutionary and functional roles of heat shock proteins including the regulation of their expression. Taken together, this study indicates that the cDNA sequences of *HSPA6* gene and its amino acid and protein structure from the Arabian camel are highly conserved and have similarities with other mammalian species. However, detailed future experiments are needed to investigate the role of camel HSPA6 in other gene regulation, function, response to environmental change, and their action at the molecular level.

## Figures and Tables

**Table 1:** Comparison and nucleotide sequence homology of the camel HSPA6 with other mammalian species.

Name	GeneBank Accession Number	cDNA Length (bp)	UTR		Total Score	Coverage %	Identity %
			5′ bp	3′ bp			
**Arabian Camel***(Camelus dromedarius)*	HQ214118	2417	136	349	2529	100	100
**Human***(Homo sapiens)*	NM_002155	2664	413	319	2523	81	89
**Sumatran orangutan***(Pongo abelii)*	XM_002809884	2398	133	334	2529	81	89
**Horse***(Equus caballus)*	XM_001488139	2124	130	62	2682	86	89
**Cow***(Bos Taurus)*	XM_589747	2210	193	85	2915	88	91
**Pig***(Sus scrofa)*	NM_001123127	2518	105	481	3025	93	91
**Mouse***(Mus musculus)*	M32218	2298	173	371	1518	75	77
**Dog***(Canis lupus familiaris)*	NM_001003067	2026	86	284	1906	79	80
**Giant panda***(Ailuropoda melanoleuca)*	XM_002931069	2356	121	309	1480	79	81

**Table 2:** Comparison and homology of the camel HSPA6 amino acid sequence, isoelectric point (pI), protein molecular weight and estimate charge at pH with other mammalian HSPs.

Name	Protein Accession Number	Amino Acid Residues	Total Score	Coverage %	Identity %	pI	Molecular Weight	Estimate Charge at pH
**Arabian Camel***(Camelus dromedarius)*	ADO12067	643	1252	100	100	6.00	70.5	−9.0
**Human***(Homo sapiens)*	NP_002146	643	1227	100	94	6.13	71.0	−7.3
**Sumatran orangutan***(Pongo abelii)*	XP_002809930	643	1252	100	94	5.86	70.9	−9.5
**Horse***(Equus caballus)*	XP_001488189	643	1217	100	93	6.20	70.8	−7.0
**Cow***(Bos Taurus)*	XP_589747	643	1244	100	94	6.04	71.0	−8.3
**Pig***(Sus scrofa)*	NP_001116599.1	643	1251	100	94	6.06	71.1	−7.5
**Mouse***(Mus musculus)*	AAA74906	641	1077	100	80	6.12	70.7	−6.0
**Dog***(Canis lupus familiaris)*	BAC79353	641	1092	99	83	6.30	70.5	−4.2
**Giant panda***(Ailuropoda melanoleuca)*	XP_002931115	641	1093	99	83	5.73	70.2	−8.4

**Table 3:** Heat shock proteins, crystal and oligomeric structures and absolute quality measures.

Name	Beta Interaction Energy (Z-score)	Pairwise Energy (Z-score)	Solvent Energy (Z-score)	Torsion Angle Energy (Z-score)	Secondary Structure Alignment (Z-score)	Solvent Accessibility Agreement (Z-score)	Total QMEANScore; Estimated Model Reliability between 0–1) (Z-score)
*Camelus dromedarius*	−263.13 (0.64)	−18120.78 (0.75)	−70.23 (0.63)	−61.42 (−2.84)	77.1% (−0.33)	76.7% (−0.81)	0.601 (1.79
*Bos Taurus*	−335.84 (1.25)	−21082.24 (1.32)	−87.35 (1.46)	−121.57 (−1.41)	78.1% (−0.13)	74.3% (−1.25)	0.606 (−1.74)
*Homo sapiens*	−206.76 (1.05)	−13292.65 (1.21)	−42.48 (0.51)	−119.06 (0.65)	83.4% (0.47)	90.5% (1.87)	0.939 (1.96)
*Mus musculus*	−594.81 (2.02)	−36711.18 (2.12)	−109.35 (0.58)	−262.22 (0.21)	85.1% (0.65)	84.5% (0.77)	0.847 (1.02)
*Canis lupus*	−720.31 (1.67)	−46429.75 (1.87)	−155.98 (1.06)	−267.45 (−0.59)	81.6% (−0.01)	74.8% (−0.92)	0.658 (−0.99)

**Table 4:** Pairwise alignment between prediction structure of camel (HSP) and crystal (HSP) of other species.

No.	Name	Chain	Z-Score	Aligned Residues	RMSD Å	Sequence Identity%
1	*Camel + Homo sapiens*	A	49.9	380	2.6	87
2	*Camel + Mus musculus*	A	47.3	376	3.0	82
3	*Camel + Canis lupus*	A	1.7	52	3.3	8
4	*Camel + Bos taurus*	A	61.2	606	2.2	27
